# Two-Dimensional Hybrid Composites of SnS_2_ Nanosheets Array Film with Graphene for Enhanced Photoelectric Performance

**DOI:** 10.3390/nano9081122

**Published:** 2019-08-03

**Authors:** Feier Fang, Henan Li, Huizhen Yao, Ke Jiang, Zexiang Liu, Congjian Lin, Fuming Chen, Ye Wang, Lai Liu

**Affiliations:** 1 SZU-NUS Collaborative Innovation Center for Optoelectronic Science & Technology, International Collaborative Laboratory of 2D Materials for Optoelectronics Science and Technology of Ministry of Education, College of Optoelectronic Engineering, Shenzhen University, Shenzhen 518060, China; 2 College of Electronic Science and Technology, Shenzhen University, Shenzhen 518060, China; 3 School of Physics and Telecommunication Engineering, South China Normal University, Guangzhou 510006, China; 4 Key Laboratory of Material Physics of Ministry of Education, School of Physics and Engineering, Zhengzhou University, Zhengzhou 450052, China

**Keywords:** graphene/SnS_2_ heterojunction, charge separation, photodetectors

## Abstract

Two-dimensional (2D) metal dichalcogenides have attracted considerable attention for use in photoelectric devices due to their unique layer structure and strong light-matter interaction. In this paper, vertically grown SnS_2_ nanosheets array film was synthesized by a facile chemical bath deposition (CBD). The effects of deposition time and annealing temperature on the quality of SnS_2_ films was investigated in detail. By optimizing the preparation conditions, the SnS_2_ array film exhibited efficient photoelectric detection performance under sunlight. Furthermore, in order to improve the performance of the photodetector based on SnS_2_ nanosheets film, a transparent graphene film was introduced as the hole-transport layer by wet-chemical method directly transferring techniques. Graphene/SnS_2_ nanosheets array film heterojunction photodetectors exhibit enhanced photoresponsivity. The light on/off ratio of the photodetector based on graphene/SnS_2_ was 1.53, about 1.4 times higher than that of the pristine SnS_2_ array films. The improved photoresponse performance suggested that the effective heterojunction between vertical SnS_2_ nanosheets array film and graphene suppresses the recombination of photogenerated carriers. The results indicate that the graphene/SnS_2_ heterojunction photodetectors have great potential in photodetection devices.

## 1. Introduction

The unique physical and chemical properties of graphene has inspired the exploration of two-dimensional (2D) materials [1]. Graphene has a huge electrical mobility that approaches 200,000 cm^2^ V^−1^ s^−1^ and is used for electrons and hole free sheets [2]. Moreover, the absorption of a single-layer graphene is ~2.3% over a broad wavelength range [3]. As a result, graphene has been used in many electronic and optoelectronic fields, including mode-locked lasers [4,5], photodetectors [6,7,8], transistors [9,10], batteries [11], photovoltaics [12], photocatalysis [13,14], and so forth. Among these applications, photodetectors are critical optoelectronic components that convert optical radiation into electrical signal [15,16] and they have been widely used in medical analysis, astronomy [17], environmental sensing [18], and optical communication [19,20], However, single-layer graphene itself is a band-free Dirac semi-metal, which limits its application in optoelectronic devices where semiconducting properties are required [21].

Recently, the combination of graphene with various two-dimensional (2D) layered materials has proven to be an effective strategy to achieve the unique electronic and optoelectronic applications of 2D materials [22,23,24]. Among the various kinds of 2D layered metal dichalcogenides, SnS_2_ is an n-type semiconductor with a layered cadmium iodide (CdI_2_) structure. Each layer is covalently bonded by three atomic planes and separated by weak van der Waals interaction. SnS_2_ exhibits a high optical absorption coefficient and strong photoconductive properties in visible regions and this has given rise to increased interest due to its particular structure, suitable band gap (~2.2 eV), good chemical stability, low-cost and environmental friendliness [25]. Therefore, it has been widely used in many fields, such as lithium (sodium) ion batteries [26,27], solar cells [28,29], photocatalysis [30,31], field effect transistors [32,33], photodetectors [34,35,36], etc. Su et al. [37] used chemical vapor deposition (CVD) to introduce metal seeds on the substrate to achieve high-quality SnS_2_ thin film for location-selective synthesis, and the response time and quantum efficiency of the fabricated photodetector was ~5 microseconds and 11.3%, respectively. Tao et al. [38] studied a transparent polypropylene (PP) film flexible photodetector based on SnS_2_ self-assembled microsphere film by double-sided tape. The device shows a good photoresponse in the region of UV (300 nm) though to NIR (830 nm) light. However, vertical SnS_2_ nanosheets array film combined with transparent graphene film for photodetector application has rarely been investigated.

In this work, SnS_2_ film vertically grown on conductive fluorine-doped tin oxide (FTO) glass substrate was fabricated by the low-temperature CBD method. The optimal fabrication conditions for pure SnS_2_ array film was investigated. After annealing treatment at 300 °C in nitrogen atmosphere, the crystalline of SnS_2_ showed an obvious improvement. A highly transparent graphene thin film was deposited onto n-type SnS_2_ array film to form a Schottky junction. The graphene/SnS_2_ heterojunction was constructed by the direct wet-chemical transfer method. Graphene, as a hole-transport layer, promotes the charge transfer at the interface of the heterojunction. The corresponding photodetector based on graphene/SnS_2_ composite film shows enhanced and stable photoelectric performance. 

## 2. Experimental Methods

### 2.1. Synthesis of SnS_2_ Nanosheets Array Film

**Figure 1:**
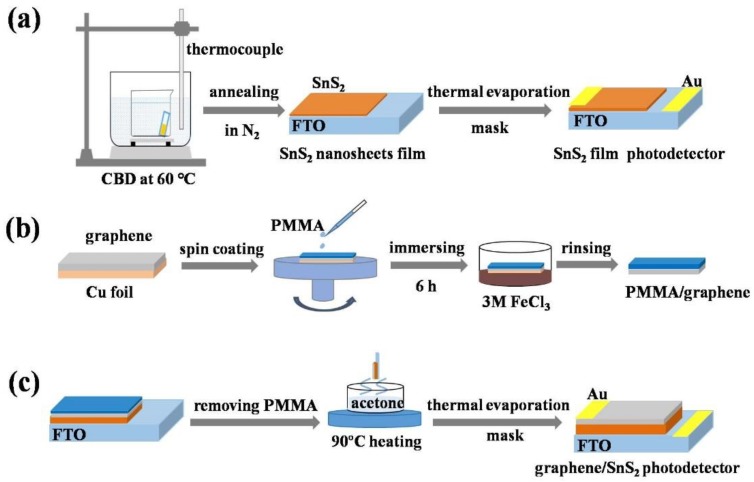
Schematic of (**a**) chemical bath deposition (CBD) process for SnS_2_ nanosheets film; (**b**) preparation of the floating graphene film; (**c**) preparation of the graphene/SnS_2_ heterojunction.

All chemicals in the experiment were of the highest purity available and were used without further purification. In this experiment, the resistivity of distilled water was 18.0 M Ω cm. FTO substrates were ultrasonically cleaned successively with acetone, isopropanol, and ethanol, followed by rinsing with deionized water and drying in the flow of N_2_. The cleaned substrates were further treated by plasma for 5 min. SnS_2_ nanosheets array film was synthesized by the CBD method, similar to that previously reported in [39]. The synthesized process of the SnS_2_ film is shown in Figure 1a. Briefly, 10 mol of SnCl_4_·5H_2_O (Aladdin, 99.995% metals basis, Shanghai, China) was dissolved in 240 mL anhydrous ethanol. The mixture was stirred for 15 min under ambient conditions before 0.15 mol thioacetamide (CH_3_CSNH_2_, Aladdin, Shanghai, China) was added. Then, the mixture was placed in a beaker of 50 mL volume. Finally, a piece of cleaned FTO substrate was placed at an angle against the wall of the beaker with the conductive side facing down. The CBD process was carried out at 60 °C in a water bath kettle for 0.5 h, 1 h and 1.5 h, respectively. When the kettle was cooled down to room temperature, the FTO substrates were removed and thoroughly rinsed with deionized water. Finally, the samples were dried in an oven at 80 °C for 6 h. To further remove any impurities on the sample surface and improve the crystallinity, the samples were annealed at 250 °C, 300 °C and 350 °C for 1 h in nitrogen (N_2_) atmosphere [40,41]. The prepared samples were dried and cleaned in ambient conditions for further characterization.

### 2.2. Preparation of Graphene/SnS_2_ Heterojunction

Graphene/SnS_2_ heterojunction was constructed by the wet-chemical transfer method [42,43], where the graphene layer was directly transferred onto SnS_2_ film. The large area of graphene films on Cu foil substrates were purchased from the Graphene Technology Corporation, China. Firstly, graphene film was delaminated from the Cu foil by the conventional polymethyl methacrylate (PMMA, Aladdin, ACS, Shanghai, China) assisted transfer method (as shown in Figure 1b) [44]. PMMA was spin-coated onto the graphene film at 2000 r/min. The PMMA/graphene/Cu foils was dried at 100 °C for 3 min, then the samples were placed in 3 M FeCl_3_ ((Aladdin, ACS, Shanghai, China) solution for 6 h to etch the Cu foil completely. The floating PMMA/graphene films were fished out and rinsed several times in deionized water. Finally, the PMMA/graphene film was picked up by FTO substrates with SnS_2_ nanosheets grown and dried in a flow of N_2_, as shown in Figure 1c. Subsequently, the whole sample was treated by acetone vapors to dissolve the PMMA by heating the acetone solution to 90 °C. The obtained graphene/SnS_2_ heterojunction films were then used for photodetector fabrication. 

### 2.3. Characterization

The morphology and microstructure of the samples were characterized by field emission scanning electron microscope (SEM) (Hitachi, Tokyo, Japan, SU8010 (MDTC-EQ-M18-01)). The composition of the crystal structure was identified by X-ray diffractometer (Rigaku, Ultima IV, Tokyo, Japan) using Cu Kα radiation (λ = 1.5418Å). UV-vis absorption was recorded with Cary 5000 UV-Vis-NIR spectrophotometer (Agilent Technologies, CA, USA) in the range of 350 nm to 800 nm. In order to investigate the photoelectric performance of the samples, Au electrodes were deposited on the samples with a shadow mask by a thermal evaporation method. The photoelectric performance of the photodetector based on the pristine SnS_2_ and graphene/SnS_2_ thin films was investigated by an electrochemical workstation (CH Instruments, model CHI 760E, Austin, TX, USA). The CHI electrochemical workstation was used to measure dark and illuminated current at a scan rate of 10 mV/s. The bias voltage was 3 V. The active area of samples was kept at 1 cm^2^ by a mask. A 500W xenon lamp was used to simulate sunlight. (Spectra Physics, CA, USA). 

## 3. Results and Discussion

**Figure 2:**
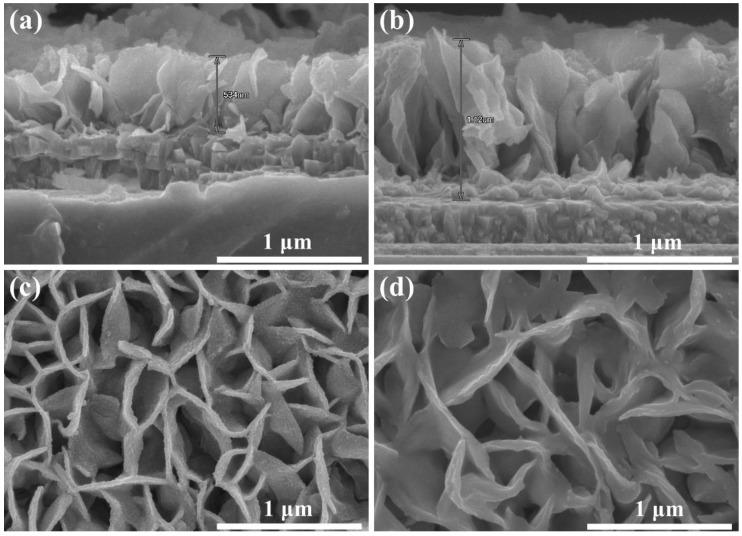
Cross-sectional view of field-emission scanning electron microscopy (FESEM) image of pristine SnS_2_ film on FTO prepared at 60 °C for (**a**) 1 h, and (**b**) 1.5 h, (**c**,**d**) are the corresponding top-view SEM images.

The vertically grown SnS_2_ array films on FTO substrate were prepared by a simple CBD method (see Experimental Methods). We investigated the density change of SnS_2_ nanosheets by changing the reaction time to obtain the optimal condition. The reaction temperature of all the samples was 60 °C. Altering the reaction time from 1 h to 1.5 h barely changed the morphology of the nanocrystalline, but the thickness of the single SnS_2_ nanosheets film increased from 534 nm to 1120 nm, as shown in Figure 2a,b. The SnS_2_ nanocrystals with non-overlapping structures were interlaced and interconnected with each other. With the extension of the reaction time, the density of ordered SnS_2_ gradually increased when the CBD time was between 0.5 h and 1 h. The top-view SEM image of pristine SnS_2_ film on FTO prepared at 60 °C for 0.5 h is shown in Figure S1 (Supplementary Materials). However, when the CBD time exceeds 1 h, the film density decreased due to the curing mechanism of the reaction (the corresponding top-view SEM images are shown in Figure 2c,d). Therefore, the optimal condition for CBD is 1 h. The effect of annealing conditions on the film density will be further explored in future work. We also measured the surface roughness of the pristine SnS_2_ and graphene/ SnS_2_ thin film on FTO prepared at 60 °C for 1 h and the AFM results are presented in Figure S2a,b (Supplementary Materials). According to the AFM results, compared with pristine SnS_2_, the average surface roughness of graphene/SnS_2_ thin film decreased from 70 nm to 45.7 nm. The results show that the sample with lower surface roughness and better compactness is prepared under this condition. In our experiment, an orange film started to peel off from the FTO substrate when the reaction time was longer than 1.5 h. The reason for the dissolution/partial peel-off might be explained by considering the reaction kinetics [45]. As the reaction time increases, the concentration of reactants in the solution decreases. When the concentration of reactants near the FTO substrate is lower than the concentration allowed by the chemical equilibrium, an opposite reaction will occur to dissociate the membrane to make up for the deficiency, which accounts for the film peel-off.

**Figure 3:**
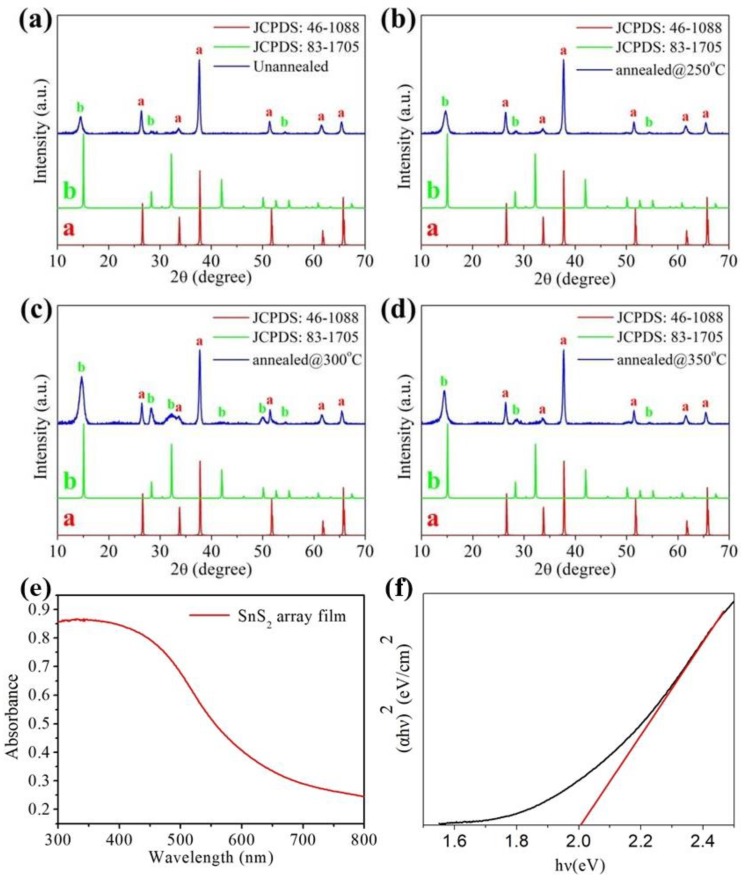
XRD pattern of pristine SnS_2_ films (**a**) unannealed, and annealed at different temperatures (**b**) 250 °C, (**c**) 300 °C and (**d**) 350 °C. (**e**,**f**) are UV–vis absorption spectra and the corresponding plots of (α hν)^2^ vs. hν of the pure SnS_2_ array film annealing at 300 °C in N_2_ atmosphere.

**Figure 4:**
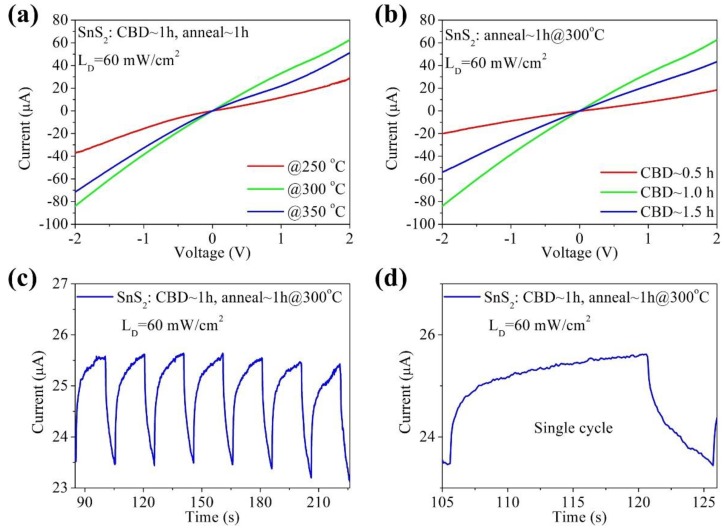
(**a**) I-V curves of photodetectors with different annealing temperatures under illumination intensity of 60 mW/cm^2^. (**b**) I-V curves of photodetectors with different CBD time. (**c**) I-t curves of photodetector with an on–off period of 20 s. (**d**) I-t curve of a single cycle of photodetector.

The crystallinity of SnS_2_ array films was characterized by X-ray diffraction. Figure 3 displays the XRD patterns of prepared samples fabricated at 60 °C and annealed at 250, 300 and 350 °C for 1 h in N_2_ atmosphere. The red curves in Figure 3 are the diffraction peaks of FTO substrate (JCPDS No. 46-1088). All of the diffraction peaks can be indexed according to the standard hexagonal structure of SnS_2_ (JPCDS No. 83-1705; space group: P/3ml (no.164); a = 0.3638 nm, c = 0.588 nm). The XRD diffraction peaks at 14.83°, 28.45°, 32.14° correspond to the (001), (100), (002) planes of the samples. No other peaks were observed in the XRD spectra at other angles, which suggests the high purity of the SnS_2_ films. The investigation also showed that no phase transition occurs during the calcining process. In addition, we used the Scherrer equation to estimate the thickness of the individual sheets that are standing up perpendicular to the substrate in the XRD data. For example, the full width at half maximum (FHWM) of the prepared samples fabricated at 60 °C for a CBD time of 1 h are 0.6217, 0.6827, 0.7382 degrees, which correspond to the XRD diffraction peaks at 14.83°, 28.45°, 32.14°, respectively. According to the Scherrer equation, the average grain thickness is 12.89 nm. Similarly, the average grain thickness of samples for 1.5 h is 14.13 nm. The data shows that the average thickness increases with the increasing reaction time. By increasing annealed temperature (Figure 3a–c), it is interesting to note that the intensity of the (100) and (002) diffraction peaks of the SnS_2_ was significantly enhanced as the annealing temperature increased, except for the sample annealed at 350 °C (Figure 3d), which indicates that the higher temperature is beneficial to obtain high crystalline samples. When the temperature of the annealing treatment increased to 350 °C, the relative intensity of the (002) diffraction peak decreased. Figure 3e shows the UV–vis absorption spectrum of pure SnS_2_ array film. The maximum peak intensity occurs at around 350 nm and the absorption edge is ~650 nm. The optical energy gap of the as-prepared SnS_2_ array film after annealing at 300 °C in N_2_ atmosphere is about 2.01 eV, as shown in Figure 3f. We further investigated the photoelectric performance of photodetectors based on pure SnS_2_ films prepared under different reaction time and annealed at different temperatures, as shown in Figure 4. The samples were illuminated under white light and the power intensity was 60 mW/cm^2^. Figure 4a is the I-V curves of the photodetector device (as shown in Figure 1a) based on the pristine SnS_2_ films annealed at different temperatures. The photocurrent shows an obvious improvement with the increased annealing temperature. However, with further increases in the annealing temperature to 350 °C, the photocurrent exhibits a decreasing tendency. This may be attributed to the deterioration in the crystallinity according to the XRD patterns shown in Figure 2d. Figure 4b shows the photoelectric performance based on the SnS_2_ films annealed at 300 °C but prepared for a different time span. The photocurrent of the sample prepared with a reaction time of 1 h is about 60 μA, higher than those of samples prepared with a reaction time of 0.5 h and 1.5 h. Based on the results, the samples used for photodetector fabrication were all prepared at 60 °C for 1 h and annealed at 300 °C in N_2_ atmosphere. Figure 4c is the corresponding photoresponse of photodetectors based on pristine SnS_2_ film prepared at optimal reaction conditions. The I-t curve was measured at a bias voltage of 3 V and the light illumination density of was 60 mW/cm^2^. Obviously, when the light is turned on (off), the photocurrent increases (decreases) sharply. It was also observed that under illumination of multiple cycles, the photocurrent and dark current did not change significantly, indicating that the performance was stable. The time response was also used to calculate the rise and decay time, which is closely related to the charge trapping/detrapping and recombination processes. The dark current of the device was large, which is probably due to the large free charge caused by the defects [46,47]. According to the individual cycle, as shown in Figure 4d, the ratio of on-off currents (I_on_/I_off_) is 1.1 and the response time (rise time/decay time) are 5.4 s and 5.1 s, respectively. This efficient photoresponse indicates that the photogenerated electrons are easily transferred from the SnS_2_ film electrodes to FTO and the as-prepared SnS_2_ film has potential application in photodetector.

**Figure 5:**
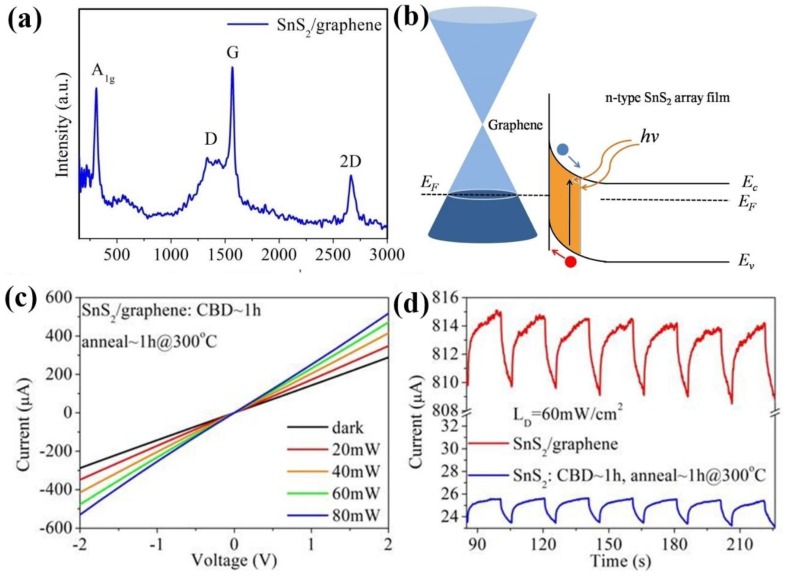
(**a**) Raman spectrum of SnS_2_/graphene composite films, (**b**) Schottky junction formed between graphene and SnS_2_ array film, (**c**) I-V curves of SnS_2_/graphene photodetector under different optical power density illumination conditions, (**d**) I-t curves of photodetectors with and without graphene under the optical power density of 60 mW/cm^2^.

Wet chemical transfer techniques were used to deposit the highly transparent graphene film on the n-type SnS_2_ array film (as shown in Figure 1, to further improve the photoelectric performance of the device. The top-view SEM image of graphene/SnS_2_ heterostructure film is shown in Figure S3 (Supplementary Materials). The composition and microstructure of the samples were further analyzed by Raman (WITec Alpha 300R). As shown in Figure 5a, the intense peak at 315 cm^−1^ is assigned to the A_1g_ mode of SnS_2_ array film. The presence of the peaks at 1352 cm^−1^, 1585 cm^−1^ and 2720 cm^−1^ corresponds to the D, G and 2D bands of graphene, respectively. The results show that the graphene/SnS_2_ composites were successfully fabricated. Figure 5c shows the I-V curves of a graphene/SnS_2_ heterostructure photodetector in the dark and at a light illumination intensity of 20 mW/cm^2^, 40 mW/cm^2^, 60 mW/cm^2^ and 80 mW/cm^2^, respectively. The asymmetry and nonlinearity of the curves indicates the non-ohmic contact between the graphene/SnS_2_ heterostructure [48,49]. As the light power density increases, the photocurrent intensity increases, indicating an effective photoresponse property. Figure 5d shows the I-t curves of the pure SnS_2_ array film and the graphene/SnS_2_ composites film under the same light illumination conditions. The graphene/SnS_2_ composites film has stable photoresponse characteristics and good reproducibility. The device based on graphene/SnS_2_ heterojunction has an increase in photocurrent of approximately 13.7 μA/cm^2^ compared with that of pure SnS_2_-based photodetectors. The on-off currents ratio (I_on_/I_off_) of graphene/SnS_2_ heterojunction is 1.53, higher than that of pure SnS_2_ film with 1.1. The results show that the high off current and low on-off current ratio may be due to the low contact resistance between graphene and SnS_2_ layers; further improvement could be made by modifying the device configuration in the future. Furthermore, the response time (rise time/decay time) of the hybrid graphene/SnS_2_ film is 5 s/4.9 s, which is superior to that of the pure SnS_2_ array film (5.4 s/5.1 s). Under a high power light illumination, due to the abundance of photogenerated electron-hole pairs, the photogenerated carriers pass through the electrode for a long time, the current slowly rises to the optimal value, and the response time is long. The enhanced photoelectric performance is because graphene as a hole transporting layer was introduced by a simple transfer method to form a Schottky junction photodetector with vertical SnS_2_ array film. As already known, graphene has a zero-band gap characteristic resulting from its valence bands exhibiting linear dispersion degeneration near Dirac point energy. Nevertheless, under a built-in electric field formed at the interface between graphene and SnS_2_ array film, graphene can be tuned to display p-type behavior, as shown in Figure 5b [50]. Once incident photons are absorbed by SnS_2_ nanosheets array film, electron–hole pairs are predominantly produced in the depletion layer. The photo-generated holes transfer swiftly to the graphene and are collected by Au electrodes. The Schottky junction is beneficial for effective charge carrier separation. Consequently, the recombination of the photogenerated electrons and holes are effectively inhibited, leading to improved photoresponse performance. Moreover, since the graphene/SnS_2_ interface has charge trap states in ambient conditions, it reduces the dark current. We repeated the measurement in the vacuum environment (INSTEC, LTS420E-PB4, CO, USA), and the current was measured by Keithley 2636B (Tektronix, OH, USA). The results are shown in Figure S4 (Supplementary Materials), which shows that the dark current in the vacuum is lower than that in the ambient, thus the photoelectric response is improved. In addition, there are many methods [51] to improve the performance of photodetectors based on 2D materials, including surface plasma enhancement, charge transfer assistance, optical waveguide integration, graphene sandwich structure and heterogeneous structure directly grown by CVD. A high concentration of defects can reduce the mobility of carriers, [52] which is an important reason for the slow response of photodetectors. Further research will focus on reducing defects in the sample and improving device fabrication processes.

## 4. Conclusions

In summary, 2D SnS_2_/graphene composites film has been fabricated in situ on FTO by using low-temperature CBD and then the direct wet-chemical transfer method. The hybrid graphene/SnS_2_ film exhibits superior photoelectric performance compared to pristine SnS_2_ nanosheets array film. The light on/off ratio for SnS_2_ nanosheets is 1.1, whereas for the graphene/SnS_2_ composite, it is 1.53. The successful demonstration of the photoelectric enhancement based on 2D graphene/SnS_2_ array film opens up new opportunities for the application of other low-temperature soluble transition metal sulfide combined with graphene in photodetectors.

## Supplementary Materials

The following are available online at https://www.mdpi.com/2079-4991/9/8/1122/s1, Figure S1: top-view SEM image of pristine SnS_2_ film on FTO prepared at 60 °C for 0.5 h, Figure S2: AFM height image of (a) pristine SnS_2_ film and (b) SnS_2_/ graphene heterostructure film on FTO prepared at 60 °C for 1 h, Figure S3: top-view SEM image of SnS_2_/ graphene heterostructure film, Figure S4: I-V curves of SnS_2_/ graphene photodetector under vacuum and ambient condition.
