# Supplementary material for: Two-Dimensional Hybrid Composites of SnS2 Nanosheets Array Film with Graphene for Enhanced Photoelectric Performance
Source: Nanomaterials (Basel). 2019 Aug 3;9(8):1122. doi: 10.3390/nano9081122 (PMC6723453; doi:10.3390/nano9081122)
Supplement: Supplementary file 1 [file nanomaterials-09-01122-s001.pdf]

Supplementary Materials

Two-Dimensional Hybrid Composites of SnS_2 Nanosheets Array Film with Graphene for Enhanced Photoelectric Performance

Feier Fang [1], Henan Li [2], Huizhen Yao [1], Ke Jiang [1], Zexiang Liu [1], Congjian Lin [1], Fuming Chen [3], Ye Wang [4] and Lai Liu [1,*]

1 SZU-NUS Collaborative Innovation Center for Optoelectronic Science & Technology, International Collaborative Laboratory of 2D Materials for Optoelectronics Science and Technology of Ministry of Education, College of Optoelectronic Engineering, Shenzhen University, Shenzhen 518060, China
2 College of Electronic Science and Technology, Shenzhen University, Shenzhen 518060, China
3 School of Physics and Telecommunication Engineering, South China Normal University, Guangzhou 510006, China
4 Key Laboratory of Material Physics of Ministry of Education, School of Physics and Engineering, Zhengzhou University, Zhengzhou 450052, China
* Correspondence: liulai_photonic@szu.edu.cn

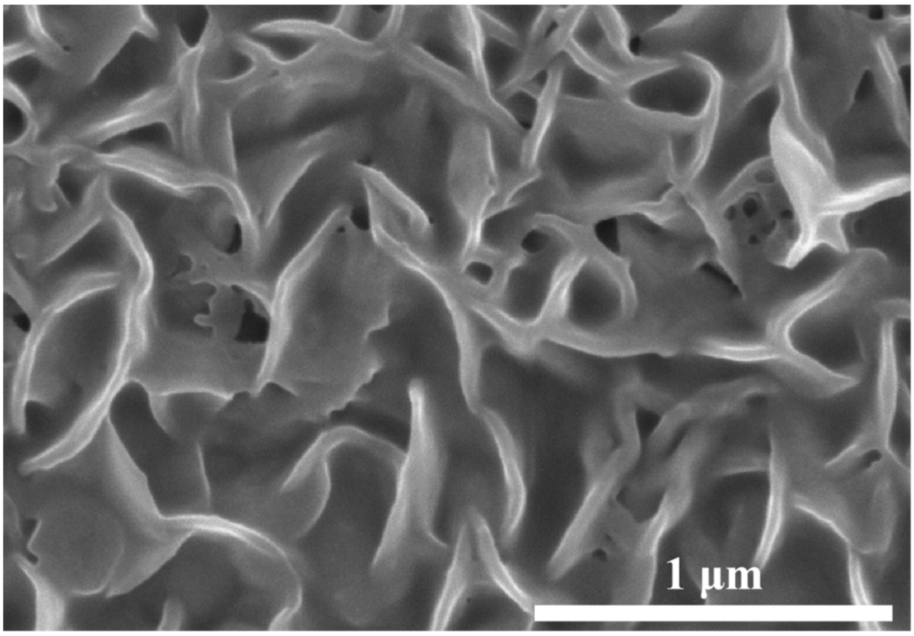

Figure 1. top-view SEM image of pristine SnS_2 film on FTO prepared at 60 °C for 0.5 h.

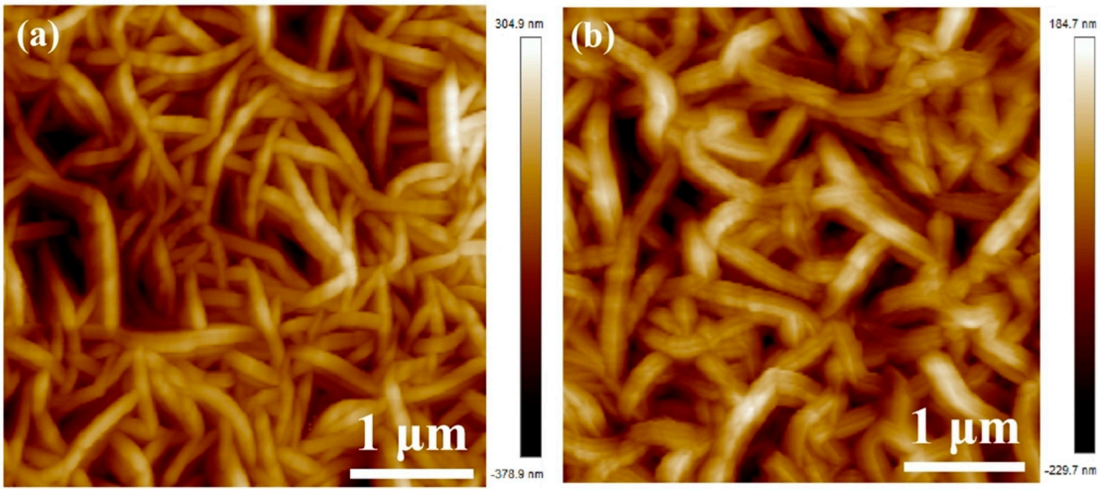

Figure 2. AFM height image of (a) pristine SnS_2 film and (b) SnS_2/ graphene heterostructure film on FTO prepared at 60 °C for 1 h.

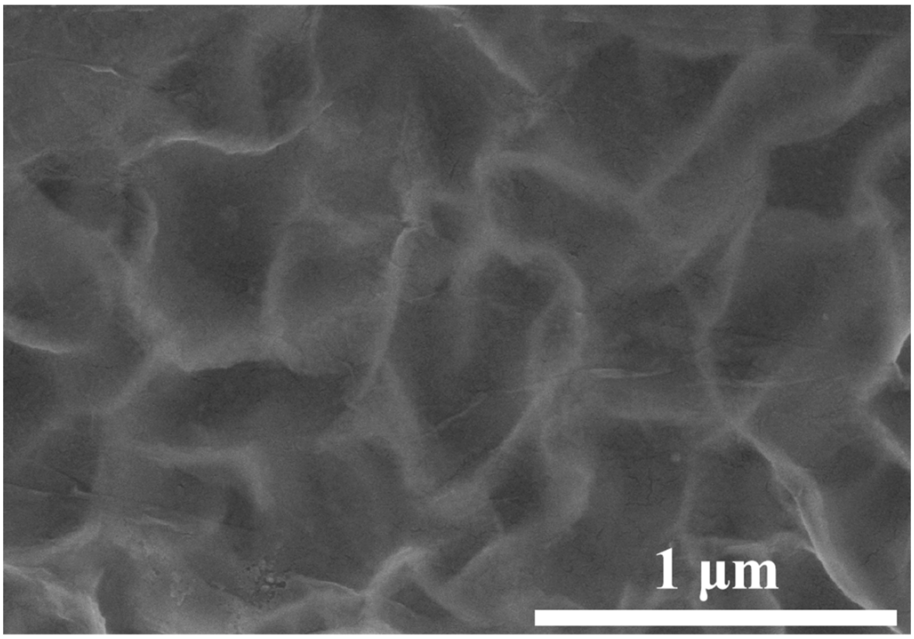

Figure 3. top-view SEM image of SnS_2/ graphene heterostructure film.

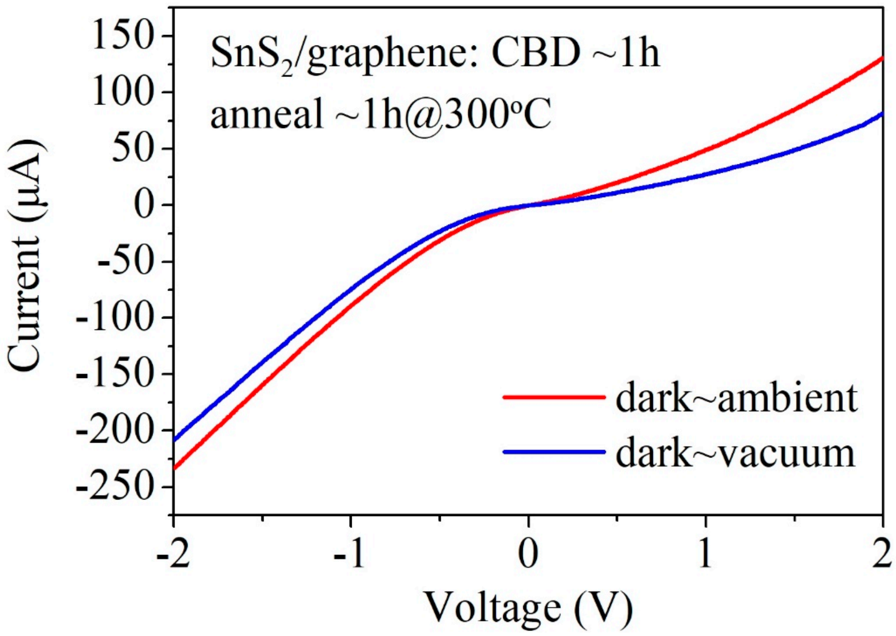

Figure 4. I-V curves of SnS_2/ graphene photodetector under vacuum and ambient condition.
